# Factors affecting the acceptance of blended learning in medical education: application of UTAUT2 model

**DOI:** 10.1186/s12909-020-02302-2

**Published:** 2020-10-16

**Authors:** Seyyed Mohsen Azizi, Nasrin Roozbahani, Alireza Khatony

**Affiliations:** 1 Medical Education and Development Center, Arak University of Medical Sciences, Arak, Iran; 2 Department of Health Education and promotion, Faculty of Health, Arak University of Medical Sciences, Arak, Iran; 3 Clinical Research Development Center of Imam Reza Hospital, Kermanshah University of Medical Sciences, Kermanshah, Iran

**Keywords:** Blended learning, UTAUT2, Medical education, Students

## Abstract

**Background:**

Blended learning is a new approach to improving the quality of medical education. Acceptance of blended learning plays an important role in its effective implementation. Therefore, the purpose of this study was to investigate and determine the factors that might affect students’ intention to use blended learning.

**Methods:**

In this cross-sectional, correlational study, the sample consisted of 225 Iranian medical sciences students. The theoretical framework for designing the conceptual model was the Unified Theory of Acceptance and Use of Technology 2 (UTAUT2). Venkatesh et al. (2012) proposed UTAUT2 as a framework to explain a person’s behavior while using technology. Data were analyzed using SPSS-18 and AMOS-23 software. Structural equation modeling technique was used to test the hypotheses.

**Results:**

The validity and reliability of the model constructs were acceptable. Performance Expectance (PE), Effort Expectance (EE), Social Influence (SI), Facilitating Conditions (FC), Hedonic Motivation (HM), Price Value (PV) and Habit (HT) had a significant effect on the students’ behavioral intention to use blended learning. Additionally, behavioral intention to use blended learning had a significant effect on the students’ actual use of blended learning (β = 0.645, *P ≤* 0.01).

**Conclusion:**

The study revealed that the proposed framework based on the UTAUT2 had good potential to identify the factors influencing the students’ behavioral intention to use blended learning. Universities can use the results of this study to design and implement successful blended learning courses in medical education.

## Background

Blended learning can be useful and effective in teaching clinical skills and medical education [1–4]. This approach is a good platform for linking theory and practice in the teaching-learning process [5]. Blended learning or hybrid learning is defined as the systematic integration of face-to-face learning and online learning [6–10]. In the blended learning environment, blending the online and face-to-face elements should be purposeful. Purposeful blending is defined as blending the tools, methods and technologies to accomplish educational purposes [11]. Some researchers use delivery medium, teaching place, teaching type and synchronicity dimensions to identify blended learning environments. Delivery medium indicates whether education is provided by the technology or the teacher. Teaching place shows whether students receive education in the classroom or online. Teaching type indicates whether content presentation (content-based education) or students’ participation (activity-based education) in the learning process is emphasized. Synchronicity refers whether students are following a group pace or individual pace [12]. Therefore, the above dimensions should be taken into account in the blended learning environments. An important point in the blended learning is that blending the potential of face-to-face and online education environments should be in line with increasing flexibility and achieving the learning goals. Hence, the learning environment should promote independence in learning, participation, interaction, self-assessment and cooperation [11]. Blended learning encourages learners to perform problem solving and confront challenges related to learning and sharing the learning experiences [13]. This approach also plays a significant role in enhancing students’ knowledge and skills and flexibility in the teaching-learning process [5]. According to the results of studies by Liu et al. (2016), Chen et al. (2020), Rowe et al. (2012), and Morton et al. (2016), blended learning has a positive effect on enhancing the learning experience and deep learning in medical education courses [13–16]. The results of a meta-analysis showed that blended learning had a positive effect on knowledge acquisition in the health sciences [17]. The findings of another study showed that blended learning enabled students to use different learning styles [5].

Most comparative studies have shown that blended learning is more effective than the face-to-face or online learning approaches [17–19]. Successful use of the blended learning approach in the curriculum requires students’ readiness to accept it. Therefore, it is important to identify the social, psychological, cultural and pedagogical factors that may influence the acceptance of blended learning. Zhao & Yuan (2010) showed that e-learning adaptability, perceived usefulness, perceived ease of use and on time teacher’s feed-back were the most important factors affecting learner satisfaction with using the blended learning approach [20]. Garcia et al. (2014) reported that the outcome expectancy, facilitating conditions and social influence had a positive impact on the behavioral intention to use blended learning [21]. The results of Tang & Chaw (2013) showed that variables such as attitudes toward online learning, study management, online interaction and learning flexibility were positively correlated with students’ readiness for blended learning [22]. Yeou (2016) reported that computer self-efficacy and perceived usefulness had an important role in the acceptance of blended learning [23]. The findings of Wu and Liu (2013) showed the positive effect of learning atmosphere, perceived enjoyment, perceived usefulness, system performance, social interaction, content specificity, and performance expectation on the students’ satisfaction with blended learning [8].

As indicated above, blended learning is an effective approach in universities [20]. Therefore, it is important to identify the key factors affecting its acceptance. Considering the paucity of knowledge about the topic of the present research, this study was conducted to identify the factors affecting students’ behavioral intention to use blended learning in medical education based on the unified theory of acceptance and use of technology 2 (UTAUT2) [24, 25]. In the following section, we will discuss the UTAUT2 and conceptual model constructs.

## Theoretical background

**Fig. 1:**
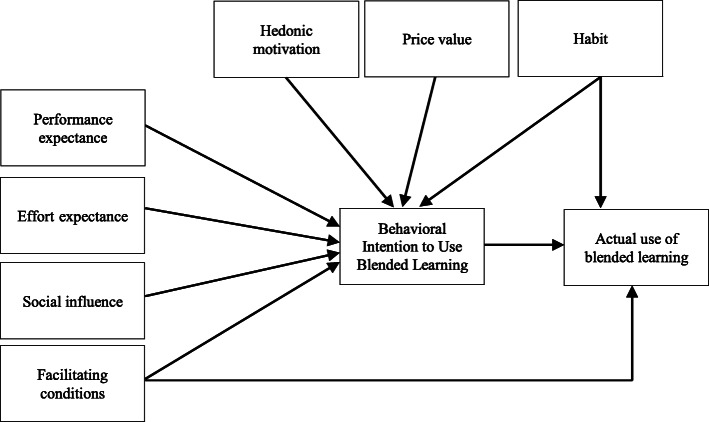
Research model, adapted from UTAUT2

The UTAUT model is one of the most important models in the field of technology adoption which has been developed by Venkatesh et al. (2003) [26]. This model is a new form of the technology acceptance model. The UTAUT model analyzes the users’ behavioral intention to use a technology [16]. The evidence shows that UTAUT explains 70% of the variance in the users’ behavioral intention to adopt a technology [23, 24]. This model consists of four main constructs regarding the intention and behavior of using a technology, including performance expectancy, effort expectancy, social influences and facilitating conditions [26]. The UTAUT2 framework was designed by Venkatesh et al. (2012) based on the original version of the UTAUT model [24]. The UTAUT2 has other constructs such as price value, hedonic motivation and habit [24, 25]. The next section describes the model constructs designed based on the UTAUT2 framework (Fig. 1).

### Performance expectancy (PE)

PE is defined as “the degree to which an individual believes that using system will help him or her to gain a profit in performance” [24]. In this study, performance expectancy refers to the learners’ belief that using blended learning is helpful. The results of some studies have indicated the positive effect of PE on the behavioral intention [27–31].

### Effort expectancy (EE)

EE is defined as “the degree of simplicity and ease of use of a system” [26]. This construct is similar to the construct of ease of use perceived in the technology acceptance model. Some studies have indicated that EE may predict behavioral intention to use the e-learning system [27, 28].

### Social influence (SI)

SI is defined as “the degree to which an individual perceives that others (such as peers and faculty members) believe he or she should use a modern system or a new approach in learning” [26]. Several studies have shown that SI has a significant influence on the behavioral intention of adopting a system [21, 32, 33].

### Facilitating conditions (FC)

FC expresses “the learner’s insights into the existence of technological and organizational infrastructure and equipment to support the use of a system” [33]. In this regard, Sattari et al. (2017) showed that FC had a significant effect on the behavioral intention and use of e-learning system [34]. However, Hoque and Sorwar (2017) reported that FC had no significant effect on the users’ behavioral intention to use the mobile health service [29].

### Hedonic motivation (HM)

HM is a new construct in the UTAUT2. This construct is defined as “the user’s pleasure of using a system” [25]. According to studies, HM is one of the important factors in predicting the intention to use e-learning and mobile learning [32, 33].

### Price value (PV)

This construct is defined as “the learners’ understanding of a trade-off between the perceived benefits of system and the monetary cost paid for the adoption of system [25]. If the benefits of acceptance of blended learning are perceived to be greater than the monetary cost, students are more likely to accept it. In this regard, Moorthy et al. (2019) reported that PV had a positive effect on the behavioral intention to use mobile learning [33].

### Habit (HT)

HT is defined as “a students’ degree of tendency to perform habitual behaviors in the teaching-learning process”. This construct is rooted in one’s past experiences [25]. A person’s favorable experiences in using a system automatically lead to the formation of a positive belief [35, 36]. Some studies have shown that HT has a positive influence on the behavioral intention [24, 32, 33].

### Behavioral intention (BI)

BI means the likelihood of a person to use a system. The actual use of a system occurs when a person intends to use it. Evidence suggest that BI has a direct impact on the actual use of system [26, 30, 36].

### Study hypotheses

H1: The seven factors of the UTAUT2 model will have a positive influence on student’s behavioral intention to use blended learning.

H2: Students’ behavioral intention to use blended learning will have a positive influence on the actual use of blended learning.

## Methods

This study was designed as a cross sectional, correlational research.

### Sample and sampling method

The study population consisted of all students of Kermanshah University of Medical Sciences (KUMS), Kermanshah, Iran. The optimal sample size was determined to be 230 people using the correlation coefficient formula (*n* = 230). Samples were selected by stratified random sampling method. The sampling stratum included the schools of KUMS. Sampling was performed in each stratum by stratified random method and using the table of random numbers. After distributing the questionnaires among the samples, 225 questionnaires were finally collected. The response rate was calculated to be 97%. The inclusion criteria comprised students’ willingness to participate in the study and studying in the second semester of the academic year 2019–2020. The exclusion criterion included the incomplete questionnaires.

### Instrument development

Data were collected by a two-part questionnaire. The first section included demographic items about gender and age. The second section included blended learning items. The questionnaire items were designed based on the UTAUT2 framework. In order to design the questionnaire items, we examined the questionnaires based on the UTAUT2 used in other studies [25, 27, 28, 37, 38]. Finally, we designed a questionnaire with 31 items and 9 constructs, including PE (5 items), EE (4 items), SI (4 items), FC (3 items), HM (4 items), PV (3 items), HT (4 items), BI (2 items) and actual use of behavior (2 items). The samples provided their responses on a 5-point Likert scale from 1 (strongly disagree) to 5 (strongly agree). The content validity and face validity of the questionnaire were evaluated by eight experts and researchers in the field of medical education, blended learning and e-learning. The validity of the final version of the questionnaire was assessed using Content Validity Indices (CVI) and Content Validity Ratio (CVR), which were 0.87 and 0.84, respectively. To determine the internal consistency, 35 questionnaires were distributed among the students. Then, the internal consistency of the questionnaire was calculated by the Cronbach’s alpha coefficient (0.942).

### Statistical analysis

The data were analyzed by SPSS (version 18.0) and AMOS (version 23.0) software. The validity of the model constructs was determined by two types of validity, including convergent validity and discriminant validity. The convergent validity was assessed using Factor Loadings (FL), Cronbach’s Alpha (CA), Composite Reliability (CR) and Average Variance Extracted (AVE). The acceptable levels were found to be greater than 0.70 for FL, CA and CR and above 0.50 for AVE [39, 40]. The discriminant validity was assessed by comparing the correlation coefficients between the constructs and the square root of the AVE.

The CR index was calculated by the formula *CR = (Σ Fl) 2 / (Σ Fl) 2 + (Σ 1-fl) 2* and the AVE index was calculated by the formula *AVE = Σ (fl2) / n* [41, 42]. The Structural Equation Modeling (SEM) was used to analyze the study hypotheses. The significant level was set at *P* ≤ 0.05.

### Procedures

This study was conducted in Kermanshah, Iran in 2019. At first, we obtained the approval of the research deputy and the National Agency for Strategic Research (NASR) in medical education at KUMS. To get the list of students, we visited the department of education of each school of KUMS. Then, the students’ list was numbered, and samples were selected based on the table of random numbers. After selecting the samples, according to their classroom schedule, the researcher attended the schools to distribute the questionnaires. The research objectives were explained to the participants and their consent to participate in the study was taken. Next, the questionnaires were given to them to complete.

### Ethical considerations

This study was conducted in accordance with the ethical considerations of the Helsinki declaration and approved by KUMS and NASR. In line with ethical requirements, the objectives of the study were explained to the participants. Furthermore, written informed consent was obtained from all of them.

## Results

The demographic characteristics of the students are given in Table 1. Of the 225 students participating in the study, 55% were female and 45% were male. The majority of students were in the age range of 20–22 (*n* = 121, 53.8%). Their mean age was 23 years. (Table 1).

**Table 1:** Demographic characteristic of respondents

Variable		N (%)
Gender	Male	45%
	Female	55%
Age (years old)	20–22	121 (53.8%)
	23–25	89 (39.6%)
	26≤	15 (6.7%)

The results of convergent validity are presented in Table 2. In order to evaluate convergent validity, the FL, CA, CR and AVE values were calculated. FL in all items were higher than 0.70. For all constructs, the CA reliability coefficients were higher than 0.70. In addition, the CR and AVE indexes were above 0.70 and above 0.50 for all constructs, respectively (Table 2). According to these results, convergent validity was at an optimal level. The results of discriminant validity are presented in Table 3. This index was evaluated using the square roots of the AVE. If the square root of the AVE of a factor is greater than the correlation coefficient of the factor, it can be stated that the questionnaire has good discriminant validity [41, 42]. In this study, the results showed that the questionnaire had excellent discriminant validity (Table 3).

**Table 2:** Measurement model results

Construct	Item	Factor Loading	Cronbach’s alpha	CR	AVE
Performance expectancy (PE)	PE1	0.87	0.95	0.931	0.840
	PE2	0.91			
	PE3	0.95			
	PE4	0.92			
	PE5	0.93			
Effort expectancy (EE)	EE1	0.84	0.88	0.893	0.678
	EE2	0.81			
	EE3	0.92			
	EE4	0.71			
Social influence (SI)	SI1	0.87	0.91	0.92	0.748
	SI2	0.95			
	SI3	0.87			
	SI4	0.76			
Facilitating conditions (FC)	FC1	0.86	0.81	0.870	0.692
	FC2	0.88			
	FC3	0.75			
Hedonic motivation (HM)	HM1	0.84	0.82	0.842	0.572
	HM2	0.75			
	HM3	0.73			
	HM4	0.70			
Price value (PV)	PV1	0.94	0.92	0.935	0.828
	PV2	0.89			
	PV3	0.90			
Habit (HT)	HT1	0.86	0.90	0.902	0.698
	HT2	0.86			
	HT3	0.85			
	HT4	0.77			
Behavioral intention (BI)	BI1	0.78	0.82	0.835	0.718
	BI2	0.91			
Use behavior (UB)	UB1	0.71	0.74	0.752	0.604
	UB2	0.84

**Table 3:** Discriminant validity results

Construct	PE	EE	SI	FC	HM	PV	HT	BI	UB
PE	0.916								
EE	0.227	0.823							
SI	0.272	0.308	0.864						
FC	0.282	0.140	0.204	0.831					
HM	0.191	0.878	0.262	0.151	0.756				
PV	0.217	0.182	0.053	0.130	0.160	0.909			
HT	0.232	0.607	0.213	0.077	0.550	0.162	0.835		
BI	0.320	0.701	0.310	0.251	0.726	0.217	0.486	0.847	
UB	0.174	0.623	0.241	0.086	0.670	0.096	0.439	0.703	0.777

**Fig. 2:**
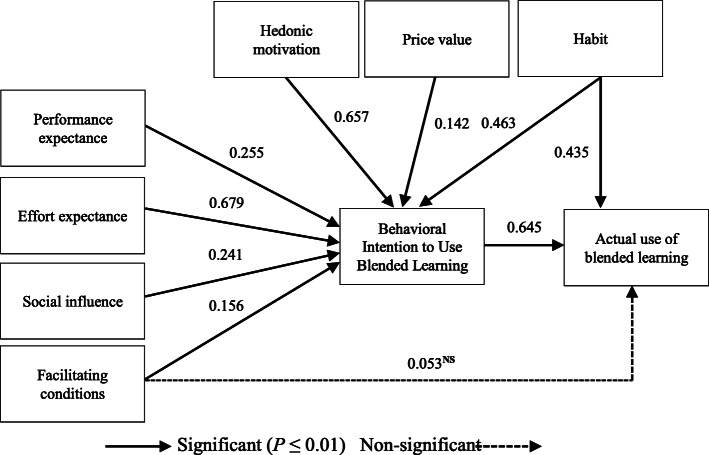
Structural model results

The results of the structural model test are presented in Fig. 2. The PE (β = 0.225, *P* ≤ 0.01), EE (β = 0.679, P ≤ 0.01), SI (β = 0.241, P ≤ 0.01), FC (β = 0.156, P ≤ 0.01), HM (β = 0.657, P ≤ 0.01), PV (β = 0.142, P ≤ 0.01) and HT (β = 0.463, P ≤ 0.01) constructs had a significantly positive effect on the students’ behavioral intention to use blended learning. HT had a significantly positive effect on the students’ use of blended learning (β = 0.435, P ≤ 0.01). In this model, FC had no significant effect on the students’ use of blended learning. Further, the students’ behavioral intention to use blended learning had a significantly positive effect on the actual use of blended learning (β = 0.645, P ≤ 0.01) (Fig. 2).

## Discussion

This study examined the factors affecting the acceptance of blended learning in medical education based on the unified theory of acceptance and use of technology 2 (UTAUT2) framework.

The results revealed that performance expectancy (PE) had a significantly positive effect on the students’ behavioral intention to use blended learning. This finding was in line with the findings of Hoque and Sorwar (2017) and Suki and Suki (2017) [29, 38]. Additionally, Abdekhoda et al. (2016) reported that PE had a significantly direct effect on the use of e-learning [27]. Therefore, the use of blended learning system in medical education is useful and valuable.

Our study showed that effort expectancy (EE) had a significantly positive effect on the students’ behavioral intention to use blended learning. The results of other studies confirm this finding [27–29]. In this regard, results of a study revealed that EE had a significant effect on the use of animation and storytelling [38]. Accordingly, the teaching-learning process seems to be easier in a blended learning environment.

Social influence (SI) had a significantly positive effect on the students’ behavioral intention to use blended learning. This finding was consistent with the results of other studies [21, 29, 34, 38]. Similarly, Ain et al. (2016) reported that SI had a positive effect on the use of learning management system [43]. The university management system and the attitudes of the faculty members and students are among the factors that shape the social and cultural atmosphere of the university. We believe that the socio-cultural atmosphere can play a role in supporting and encouraging students to use the blended learning system.

Facilitating Conditions (FC) had a significant effect on the students’ behavioral intention to use blended learning. However, this construct had no significant effect on the students’ actual use of blended learning. The results of the studies by Abdekhoda et al. (2016) and Tarhni et al. (2017) [27, 28] also showed that FC had no effect on the acceptance of e-learning. In our opinion, software and hardware infrastructures play a very important role in the students’ behavioral intention to use of blended learning. Students should be provided with technological support such as high-speed internet and advanced computers to use the blended learning system. They should also have access to sufficient resources and information about blended learning. Effective and successful implementation of the blended learning system requires good governance in higher education and advanced Information and Communication Technology (ICT) infrastructure [44]. Hedonic motivation (HM) had a significantly positive impact on the students’ behavioral intention to use blended learning. This was in line with the findings of Tarhni et al. (2017) and Moorthy et al. (2017) [28, 33]. Moorthy et al.’s (2017) study showed that HM had a significant correlation with behavioral intention to use mobile learning [33]. Enjoyable learning experiences are an important factor in using blended learning. A user-friendly environment and e-content have a significant impact on creating enjoyable learning experiences [45]. Therefore, educational designers should pay attention to these features.

This study found that price value (PV) had a significantly positive impact on the students’ behavioral intention to use blended learning. According to our findings, from the perspective of the Iranian students, inexpensive access to the blended learning materials and the use of the Internet were important factors involved in the acceptance of blended learning. This result is consistent with the findings of the study of Moorthy et al. (2019) in the Malaysian students [33]. Tarhini et al. (2017) conducted a research on the acceptance of e-learning by English students [28]. Their study showed that PV had no effect on the acceptance of e-learning. The results of another study [46] showed that from the perspective of American and Qatari students, PV had no effect on the acceptance of e-learning. We believe that different economic and social conditions in the developed and developing countries affect the students’ views about this factor.

According to the findings of this study, habit (HT) had a positive effect on the students’ intention to use blended learning. In addition, HT had a positive effect on the students’ actual use of blended learning. This was in line with the results of Tarhni (2017) and Moorthy et al. (2017) [28, 33]. Venkatesh et al. (2012) revealed that the routine use of a technology had a significant effect on its adoption [24]. Overall, our study showed that the behavioral intention to use blended learning had a significant influence on the students’ actual use of blended learning. Our results were consistent with the findings of other studies [29, 38, 47, 48]. Behavioral intention predicted the actual use of blended learning. The actual use of blended learning also depended on the students’ behavioral intention to use it.

In summary, our findings showed that Iranian students were willing to use the blended learning system to improve the quality of their learning experiences. According to the UTAUT2 framework, studies have been conducted on students from other countries, including Qatar and the United States [46], the United Kingdom [28], Malaysia [33], Jordan [49], China [50] and Spain [51]. We believe that students in the developed and developing countries have different economic, social and cultural backgrounds. These different conditions may play an important role in the students’ intention to adopt a new learning system. In Iran as a developing country, blended learning is an emerging approach in its universities. Therefore, it is recommended to conduct other studies on blended learning.

This study had some limitations. A self-reporting scale was used to assess the behavioral intention to use blended learning. This method of data collection might have affected the accuracy of the results. Qualitative methods are suggested to be used in the future studies. In this study, we identified some factors affecting the acceptance of blended learning using the UTAUT2 framework. Future studies are suggested to investigate the influence of other factors such as attitude toward blended learning, technology anxiety, experience, self-efficacy, compatibility and resistance to change on the behavioral intention to use blended learning. In addition, it is necessary to analyze the role of moderator variables such as sex, age, experience and voluntariness in the future studies. The present study was conducted on the students of neo university, so the findings may not be generalizable to other universities. Given the infrastructure differences between universities in technological and pedagogical domains, we suggest that this study be conducted in other universities.

## Conclusion

The results of the current study revealed that the model designed based on the unified theory of acceptance and use of technology 2 (UTAUT2) has good potential for identifying the factors influencing the use of blended learning in medical education. The performance expectancy, effort expectancy, social influence, facilitation conditions, hedonic motivation, price value, and habit constructs had a significantly positive effect on the students’ intention to use blended learning. A review of the literature showed that the findings of the study were consistent with some studies. This study provides a good reference for further research on blended learning in medical education. The results showed that individual-psychological factors such as performance expectancy and effort expectancy, organizational factors such as facilitation conditions and social factors such as social influence in the UTAUT2 have a significant effect on the students’ behavioral intention to use blended learning. Providing a social context and organizational support and changing the students’ psychological attitudes toward new learning approaches are essential steps involved in the successful implementation of the blended learning system.

## Data Availability

The datasets and analyzed during the present research are available from the corresponding author on reasonable request.

## Abbreviations

UTAUT2: Unified Theory of Acceptance and Use of Technology 2
PE: Performance expectance
EE: Effort expectance
SI: Social influence
FC: Facilitating conditions
HM: Hedonic motivation
PV: Price value
HA: Habit
BI: Behavioral intention
UB: Use behavior
KUMS: Kermanshah University of Medical Sciences
SPSS: Statistical Package In Social Sciences
AMOS: Analysis of Moment Structures
FL: Factors loadings
CR: composite reliability
AVE: average variance extracted
CA: Cronbach’s alpha
CVI: content validity indices
CVR: Content Validity Ratio
